# Value of serum CRP and IL-6 Assays combined with Pancreatitis activity scoring system for assessing the severity of patients with acute pancreatitis

**DOI:** 10.12669/pjms.40.1.7550

**Published:** 2024

**Authors:** Fang Xu, Xin Hu, Shu-ling Li

**Affiliations:** 1 Fang Xu, Department of ICU, Affiliated Hospital of Hebei University, Baoding, Hebei, 071000, P. R. China; 2 Xin Hu, Electrocardiogram Room, Affiliated Hospital of Hebei University, Baoding, Hebei, 071000, P. R. China; 3 Shu-ling Li, Department of Critical Care Medicine, Baoding Lianchi District People’s Hospital, Baoding, Hebei, 071000, P. R. China

**Keywords:** Acute pancreatitis, PASS score, IL-6, CRP, Diagnosis

## Abstract

**Objective:**

To evaluate the accuracy of serum CRP and IL-6 assays combined with the pancreatitis activity scoring system (PASS) in assessing the severity of patients with acute pancreatitis (AP).

**Methods:**

This was a retrospective study of 223 patients with AP admitted to Baoding Lianchi District People’s Hospital between February 2021 and 2023. They were classified into three categories: mild AP (MAP), moderate severe AP (MSAP) and severe AP (SAP). The differences, accuracy and sensitivity of the individual assays, and the three in combination, were compared and analysed in the three groups.

**Results:**

PASS scores, IL-6 and CRP levels were significantly higher in the SAP and MSAP groups compared to those in the MAP group, with statistically significant differences between the three groups. Multi-factorial logistic regression analysis suggested that PASS, IL-6 and CRP were correlated indicators of AP severity. The combination of the three assays was higher than that of the PASS score, IL-6 and CRP alone, suggesting optimal diagnostic efficacy when the three assays were combined. Moreover, the levels of PASS score, IL-6 and CRP showed a positive correlation with the degree of disease severity.

**Conclusions:**

The serum CRP, IL-6 and PASS scores were significantly elevated in AP patients and showed a positive correlation with disease severity, all of which are beneficial for the diagnosis of AP. PASS is superior to CRP and IL-6 in the assessment of AP. The combination of the three assays can achieve a far superior diagnostic efficacy to that of the individual index assays.

## INTRODUCTION

Acute pancreatitis (AP) is a common high-risk disease that poses a great threat to patients’ life and health in clinical practice. According to a survey, about 1 in 5 patients with pancreatitis have severe AP (SAP), which has a mortality rate of more than 20%.^1^ In the 1980s, most AP cases died in the early stages of the disease. Recent advances in surgical pancreatic treatment have seen an increase in the AP cure rate, but the overall mortality rate is still as high as 17%.^2^ Accurate assessment and treatment of SAP patients is a key component in the effective control of disease progression and in improving the prognosis.^3^ The current classification system for AP comprises three categories^4^ decided at the Acute Pancreatitis Classification Conference in Atlanta in 1992: mild acute pancreatitis (MAP), moderate severe acute pancreatitis (MSAP) and SAP. This was followed by a series of systems for prognostic assessment of AP according to the 1992 Atlanta classification, such as the Ranson score, Acute Physiology and Chronic Health Status Evaluation II (APACHE II) and Bedside Index for Severity in AP (BISAP), all of which showed low specificity and sensitivity. Currently, there is a lack of clinical systems for prognostic assessment of new classification systems. However, the pancreatitis activity scoring system (PASS), recently developed by several pancreatitis experts, has been shown to be useful in the assessment of MSAP^5^, although there are few clinical studies of it. Given that AP is accompanied by a significant systemic inflammatory response^6^, we investigated the value of serum CRP and IL-6 assay combined with PASS in assessing the severity of AP patients.

## METHODS

This was a retrospective study. Two hundred and twenty-three patients with AP admitted to Baoding Lianchi District People’s Hospital between February 2021 and February 2023 were identified and classified into either the MAP, MSAP or SAP group according to the 2012 edition of the Revised Atlanta Classification of Acute Pancreatitis. There were 86, 77 and 60 cases, respectively. Patient data, including demographic data, were retrieved from the electronic medical record system. The MAP group and the MSAP group were combined as the non-SAP group. No significant difference was observed in the comparison of general data between the three groups (Table-I).

**Table-I:** Comparative analysis of general information of the three groups (*χ̅*±*S*).

Indicator	MAP group	MSAP group	SAP group	F	p
n	86	77	60		
Male (cases %)	56 (65.12%)	51 (66.23%)	42 (70.00%)	0.398	0.819
Age (years old)	57.53 ± 9.82	56.83 ± 9.70	57.62 ± 9.26	0.149	0.862
Hypertension (cases %)	22 (25.58%)	21 (27.27%)	18 (30.00%)	0.348	0.840
Diabetes mellitus (cases %)	28 (32.56%)	23 (29.87%)	21 (35.00%)	0.411	0.814
BMI (kg/m^2^)	24.36 ± 2.19	23.93 ± 2.58	24.10 ± 2.42	0.660	0.518

P>0.05.

### Ethical Approal

The study was approved by the Institutional Ethics Committee of Affiliated Hospital of Hebei University (No.: HDFYLL-KY-2023-009; date: 19 January 2023), and written informed consent was obtained from all participants.

Four mililiter of venous blood was drawn from all patients after admission, and the specimens were routinely separated at high speed. The relevant tests were completed within one hour of serum collection. Serum IL-6 and CRP levels were measured by the immunoturbidimetric method using a HITACHI 7600 automatic biochemical analyser.

### Inclusion criteria:

- Aged <70 years, time from onset to admission ≤24 h.
- Hospitalisation time >48 h, the diagnostic criteria for AP were met.^7^
- The patient and family were informed about this study, the informed consent form was signed and clinical data were complete.

### Exclusion criteria:

- Concurrent heart, lung, liver or kidney diseases or malignant tumours.
- Pre-existing haematologic disorders or other pancreatic diseases
- Abnormal immune function or chronic inflammatory diseases.
- A history of pancreatic cancer or pancreatitis surgery.
- Recent use of drugs, such as immunosuppressants and hormones, that could affect the outcome of the study.
- Pregnant or breastfeeding women.
- Mental illness or other reasons that could prevent cooperation during the study.

### PASS criteria

(1) Organ failure (1 point for each system of respiratory, circulatory and renal, and cumulative for multiple system failure) × 100. (2) Inability to tolerate solid food (1 point for intolerance, 0 point for tolerance) × 40. (3) SIRS (1 point for each abnormal diagnostic criterion, cumulative for multiple abnormalities) × 25. (4) Abdominal pain (0–10 points on the pain rating scale) × 5. (5) Use of intravenous pain medication (converted to 1 mg of morphine equivalent) × 5. The PASS score is obtained by multiplying the score for each item by its weight number and adding the five scores. The higher the score, the more severe the disease; a PASS score of >90 indicates AP and a score of >140 indicates MSAP.^8^

### Observation indicators

The differences in PASS scores and the levels of IL-6 and CRP in the three groups were compared and analysed, and the accuracy and sensitivity of the combined assay of PASS score, IL-6 and CRP, and those of the individual assays were compared. The maximum follow-up time for patients in both groups was six months.

### Statistical Analysis

All data in this study were processed using SPSS 20.0 statistics software. Measurement data were expressed as (*χ̅*± S), and count data were compared as an absolute value or component ratio. The t-test was employed for comparison between groups, and the χ^2^ test was used for comparison of rates. Logistic regression analysis was employed to analyse the area under the curve (AUC) and the optimal diagnostic threshold for PASS, IL-6, CRP and the combined assay. Moreover, the AUC and optimal diagnostic threshold of each indicator were calculated for predicting SAP. The Pearson correlation coefficient was used to express the correlation between PASS scores and inflammatory indexes, with *P* < 0.05 indicating a statistically significant difference.

## RESULTS

The PASS score, IL-6 and CRP levels were significantly higher in the SAP and MSAP groups than in the MAP group, with statistically significant differences among the three groups (*P* = 0.000) (Table-II). Logistic regression analysis was performed with the disease severity as the dependent variable and PASS score, IL-6 and CRP as independent variables. PASS (*P* = 0.001), IL-6 (*P* = 0.000), PCT (*P* = 0.000) and CRP (*P* = 0.019) were independent risk factors for predicting AP severity (Table-III).

**Table-II:** Comparative analysis of the differences in PASS score, IL-6, and CRP levels among the three groups (*χ̅*±*S*).

Indicator	MAP group	MSAP group	SAP group	F	p
n	86	77	60		
PASS score	121.28 ± 8.48	155.39 ± 12.95	173.08 ± 9.79	463.013	0.000
CRP (mg/L)	25.43 ± 5.72	28.63 ± 5.55	32.76 ± 5.48	30.315	0.000
IL-6 (ng/ml)	9.26 ± 2.23	11.32 ± 2.34	15.83 ± 2.32	147.462	0.000

P < 0.05.

**Table-III:** Logistic regression analysis of PASS score, IL-6 and CRP for AP.

Item	β value	SE value	Waldχ^2^	P value	95.0%CI
PASS	0.253	0.038	44.920	0.000	0.179~0.327
CRP	0.132	0.049	7.273	0.007	0.036~0.227
IL-6	0.982	0.175	31.634	0.000	0.640~1.324

**Fig.1:**
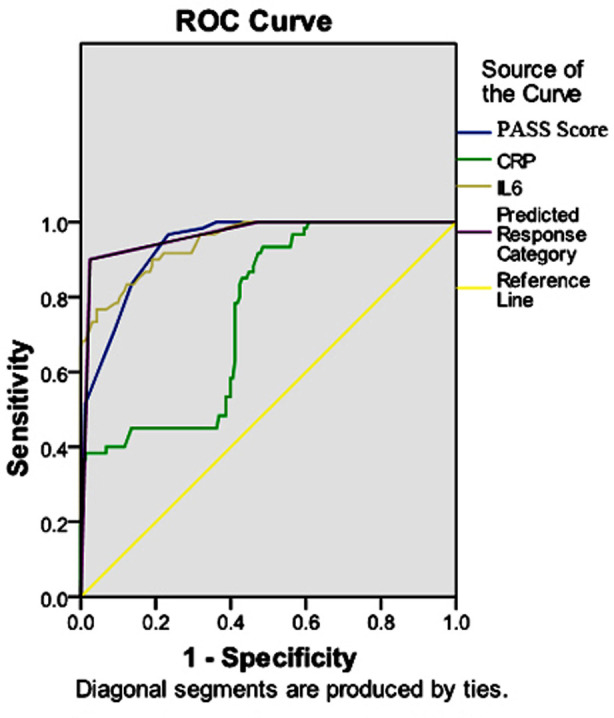
Predictive ROC curve of PASS score, IL-6, CRP and the combined assay of the three for AP.

The predictive ROC curve for the PASS score, IL-6, CRP and the combined three assays for AP showed that the AUCs of the PASS score, IL-6, PCT and CRP in predicting AP were 0.939, 0.752, 0.959 and 0.947, respectively. The AUC of the combined three assays was 0.964, with an optimal sensitivity of 90.00% and specificity of 97.50%, which was higher than that of the separate assays, suggesting that the combined assay had optimal diagnostic efficacy (Table-IV, Fig.1).

**Table-IV:** Diagnostic significance of PASS score, IL-6, CRP and the combined assay of the three in AP.

Indicator	Critical value	Sensitivity	Specificity	AUC	Youden index	95.0%CI	P value
PASS score	157.5	0.967	0.767	0.939	0.734	0.911~0.968	0.000
CRP (mg/L)	26.2	0.933	0.515	0.752	0.449	0.684~0.820	0.000
IL-6 (ng/ml)	14.0	0.767	0.957	0.947	0.724	0.918~0.977	0.000
Combined assay		0.900	0.975	0.964	0.875	0.938~0.990	0.000

Correlation analysis suggested that the PASS score, IL-6 and CRP levels all showed a positive correlation with increasing AP severity (Table-V). This indicates that the three assays have a synergistic effect in determining AP severity.

**Table-V:** Correlation analysis of AP severity with PASS score, IL-6, and CRP levels.

	PASS score		CRP (mg/L)		IL-6 (ng/ml)	

	r value	P value	r value	P value	r value	P value
AP	0.884	0.000	0.464	0.00	0.738	0.000

## DISCUSSION

This study showed that the PASS score was positively correlated with the severity of the disease; moreover, the AUC of PASS for the diagnosis of SP was 93.9% with a sensitivity of 96.70% and specificity of 76.70%, which was similar to the findings of Wu et al.^9^ However, some scholars^10^ believe that only patients with critical illnesses such as severe pancreatitis and sepsis benefit from the specificity and sensitivity of PASS, while the sensitivity is only 44% in the early stage of the disease. Therefore, more indicators are needed for a combined assay to further increase the clinical value of PASS.

AP, a common acute abdominal condition in general surgery, varies in severity from MAP to SAP. SAP is an aggressive disease characterised by rapid progression, various complications and a high mortality rate.^11^ The pathogenesis of AP remains unknown, although there are three recognised mechanisms: pancreatic enzyme activation causing pancreatic self-digestion, excessive activation of leukocytes and pancreatic blood circulation disorders. These mechanisms interact to promote the development of AP.^12^ Activation of pancreatic enzymes causes the breakdown of necrotic tissue in the pancreas to produce vasoactive substances, such as vasodilators, kinin and prostaglandin. Coupled with extensive fluid exudation around the pancreas, and a sharp decrease in blood volume and blood pressure, adverse consequences such as circulatory dysfunction and kidney damage will be further triggered. In addition, necrotoxin causes damage to other organs, such as the heart, liver and lungs and disrupts other functions. Damaged pancreatic tissue may also become antigenic or an inflammatory irritant to activate macrophages and release inflammatory mediators, resulting in a cytokine storm and immune dysfunction. An excessive systemic inflammatory response further aggravates pancreatic tissue lesions. Without timely corrective measures being made for AP, there is a high risk of secondary systemic inflammatory syndromes (sIRs) and multiple organ failure syndromes (MODs), leading to patient death.^13^

Given the rapid development of AP and its associated morbidity, it is of great importance to determine its severity in clinical practice. The three most commonly used scoring systems, the Acute Physiology and Chronic Health Evaluation II (APACHEII), the Ranson score for predicting the prognosis of AP and the BISAP, are useful for the evaluation of prognosis in AP. However, they are considered by some scholars to be difficult to calculate, in addition to being less than satisfactory in their specificity and sensitivity.^14^ Some patients with an APACHEII score of ≥8 do not progress to ASP, whereas a proportion of patients with lower scores do progress. In view of the high mortality rate and reduced healing of ASP, it is imperative to improve the specificity and sensitivity of its diagnosis.

The PASS, developed by several pancreatitis experts in recent years, has been shown to be valuable for the assessment of moderate to severe AP in preliminary studies.^15^ It has also been shown to be useful for the dynamic assessment of the prognosis of AP and has clinical value by virtue of its simplicity and ease of use.^16^

Effective assessment and diagnosis of AP and the adoption of diagnostic and therapeutic measures are crucial to control the disease and improve its prognosis. In terms of the pathological and physiological mechanisms of AP, activation of pancreatic protease leads to self-digestion of pancreatic tissue and activation of a large number of inflammatory mediators when necrotic tissue enters the bloodstream, triggering a waterfall-like cascade of inflammatory factors in the body, which in turn produces a systemic inflammatory response syndrome and affects multiple organ functions.^17^ It was concluded that the level of inflammatory factors in patients increases rapidly with the appearance of inflammatory response and correlates with the severity of the disease.^18^ Tazeoğlu et al.^19^ concluded that hypersensitivity reactive protein C (CRP), as an acute phase response protein, can effectively respond to the degree of AP inflammation. As one of the early diagnostic bases of AP, CRP plays a role in activating complement, as well as regulating immunity. In normal patients, serum CRP levels are low and often rise to a peak within a short period of time (1-2 days) in response to bacterial infection and inflammation. As the disease improves, serum levels rapidly return to normal. Serum interleukin 6 (IL-6), an important inflammatory factor in the occurrence and development of AP, is mainly produced by monocytes under the induction of IL-1 and TNF, which can produce a variety of acute reactive proteins.^20^ The levels of these acute reactive proteins directly reflect the degree of pancreatic injury in AP patients. Rasch et al.^21^ revealed that serum CRP and serum IL-6 were significantly higher in patients with SAP. It was confirmed in our study that IL-6 and CRP levels were significantly higher in the SAP and MSAP groups compared to the MAP group, with statistically significant differences between all three groups (P = 0.000). Multi-factorial logistic regression analysis suggested that PASS (P= 0.000), IL-6 (P = 0.007) and CRP (P = 0.000) were correlation indicators of the severity of AP. The levels of PASS score, IL-6 and CRP increased with the degree of severity of the disease, showing a positive correlation. Under the combined assay of the three, the area under the curve was 99.4% and the specificity was 97.50%, both of which were higher than that of PASS score, IL-6 and CRP alone, suggesting the optimal diagnostic efficacy when the three methods were combined.

### Limitations

This is a retrospective and single-center study that may lead to selection bias. In addition, a smaller sample size was included and some patients with incomplete clinical data were not included in the study, which may have led to incomplete data analysis. In response to this, more samples will be included and follow-up will be extended in future clinical work, and prospective and multicenter studies will be conducted progressively to verify the findings of this study.

## CONCLUSION

In summary, serum CRP, IL-6 and the PASS score are significantly elevated in AP patients and show a positive correlation with disease severity. This is beneficial for the diagnosis of AP, and the combination of the three assays can achieve a diagnostic efficacy far superior to that of each individual index assay. Given its simplicity and convenience, the combined assay of serum CRP, IL-6 and the PASS score provide a means of assessing the severity of disease in AP patients, which is worthy of wide clinical application.

### Authors’ Contributions:

**FX** carried out the studies, participated in collecting data, drafted the manuscript, are responsible and accountable for the accuracy and r integrity of the work.

**XH** performed the statistical analysis and participated in its design.

**SL** participated in acquisition, analysis, interpretation of data and draft the manuscript.

All authors read and approved the final manuscript.

## References

[1] Boxhoorn L, Voermans RP, Bouwense SA, Bruno MJ, Verdonk RC, Boermeester MA (2020). Acute pancreatitis. Lancet.

[2] Gliem N, Ammer-Herrmenau C, Ellenrieder V, Neesse A (2021). Management of Severe Acute Pancreatitis:An Update. Digestion.

[3] James TW, Crockett SD (2018). Management of acute pancreatitis in the first 72 hours. Curr Opin Gastroenterol.

[4] Banks PA, Bollen TL, Dervenis C, Gooszen HG, Johnson CD, Sarr MG (2013). Classification of acute pancreatitis--2012:revision of the Atlanta classification and definitions by international consensus. Gut.

[5] Buxbaum J, Quezada M, Chong B, Gupta N, Yu CY, Lane C (2018). The Pancreatitis Activity Scoring System predicts clinical outcomes in acute pancreatitis:findings from a prospective cohort study. Am J Gastroenterol.

[6] Ołdakowska M, Ściskalska M, Kepinska M, Marek G, Milnerowicz H (2022). Association of Genetic Variants in IL6 Gene (rs1800795) with the Concentration of Inflammatory Markers (IL-6, hs-CRP) and Superoxide Dismutase in the Blood of Patients with Acute Pancreatitis-Preliminary Findings. Genes (Basel).

[7] Lee PJ, Papachristou GI (2019). New insights into acute pancreatitis. Nat Rev Gastroenterol Hepatol.

[8] Knoph CS, Cook ME, Fjelsted CA, Novovic S, Mortensen MB, Nielsen LBJ (2021). Effects of the peripherally acting μ-opioid receptor antagonist methylnaltrexone on acute pancreatitis severity:study protocol for a multicentre double-blind randomised placebo-controlled interventional trial, the PAMORA-AP trial. Trials.

[9] Wu Q, Wang J, Qin M, Yang H, Liang Z, Tang G (2021). Accuracy of conventional and novel scoring systems in predicting severity and outcomes of acute pancreatitis:a retrospective study. Lipids Health Dis.

[10] Paragomi P, Tuft M, Pothoulakis I, Singh VK, Stevens T, Nawaz H (2021). Dynamic changes in the pancreatitis activity scoring system during hospital course in a multicenter, prospective cohort. J Gastroenterol Hepatol.

[11] Mederos MA, Reber HA, Girgis MD (2021). Acute Pancreatitis:A Review. JAMA.

[12] Waller A, Long B, Koyfman A, Gottlieb M (2018). Acute Pancreatitis:Updates for Emergency Clinicians. J Emerg Med.

[13] Olson E, Perelman A, Birk JW (2019). Acute management of pancreatitis:the key to best outcomes. Postgrad Med J.

[14] Shi L, Zhang D, Zhang J (2020). Albumin-bilirubin score is associated with in-hospital mortality in critically ill patients with acute pancreatitis. Eur J Gastroenterol Hepatol.

[15] Paragomi P, Hinton A, Pothoulakis I, Talukdar R, Kochhar R, Goenka MK (2022). The Modified Pancreatitis Activity Scoring System Shows Distinct Trajectories in Acute Pancreatitis:An International Study. Clin Gastroenterol Hepatol.

[16] Yu Z, Ni Q, Zhang P, Jia H, Yang F, Gao H, Gao H (2022). Clinical utility of the pancreatitis activity scoring system in severe acute pancreatitis. Front Physiol.

[17] García Vázquez E, Martínez JA, Mensa J, Sánchez F, Marcos MA, de Roux A (2003). C-reactive protein levels in community-acquired pneumonia. Eur Respir J.

[18] Arif A, Jaleel F, Rashid K (2019). Accuracy of BISAP score in prediction of severe acute pancreatitis. Pak J Med Sci.

[19] Tazeoğlu D, Akyüz C, Gökçeimam M, Harman Kamalı G, Özsoy A, Karahan SR (2021). Effect of alpha-tocopherol and dose sensitivity on pancreatitis formation in rats with experimental pancreatitis.

[20] Paliwal A, Nawal CL, Meena PD, Singh A (2022). A Study of Procalcitonin as an Early Predictor of Severity in Acute Pancreatitis. J Assoc Physicians India.

[21] Rasch S, Sancak S, Erber J, Wießner J, Schulz D, Huberle C (2022). Influence of extracorporeal cytokine adsorption on hemodynamics in severe acute pancreatitis:Results of the matched cohort pancreatitis cytosorbents inflammatory cytokine removal (PACIFIC) study. Artif Organs.

